# First detection of bovine viral diarrhea virus using antigen capture ELISA and RT-PCR assay in cattle with respiratory and abortive issues in Morocco

**DOI:** 10.5455/javar.2025.l985

**Published:** 2025-12-25

**Authors:** Hanane Khallouki, Fatiha El Mellouli, Hasnae Zekhnini, Safaa Sabri, Houria Abdelghaffar

**Affiliations:** 1 Biotechnology, Agrifood, Materials and Environment Laboratory, Faculty of Sciences and Technology of Mohammedia, Hassan II University, Casablanca, Morocco; 2 Biodiversity, Ecology and Genome Laboratory, Faculty of Sciences, Mohammed V University in Rabat, Rabat, Morocco; 3 Immunology and Biodiversity Laboratory, Faculty of Science Ain Chock, Hassan II University of Casablanca, Casablanca, Morocco; 4 Health and Environment Laboratory, Faculty of Sciences Ain Chock, Hassan II University, Casablanca, Morocco

**Keywords:** Casablanca-Settat region, Cattle, Bovine viral diarrhea virus (BVDV), ELISA, RT-PCR

## Abstract

**Objective:**

This research assessed the prevalence of Bovine Viral Diarrhea Virus (BVDV) among cattle herds from five provinces in the Casablanca-Settat region of Morocco (Benslimane, Settat, Berrechid, El Jadida, and Sidi Bennour), which experience respiratory and reproductive disorders. Antigen capture ELISA and real-time RT-PCR were utilized for the detection of BVDV.

**Materials and Methods:**

From January 2021 to June 2025, 500 blood serum samples and 500 leukocyte samples from cattle with respiratory or abortive symptoms were collected and analyzed using an antigen capture ELISA assay and a real-time RT-PCR assay, respectively, for BVD antigens or genome sequence.

**Results:**

Of the 500 sampled cattle, 9 (1.8%) tested positive, with all 9 results being actively infected with BVDV confirmed via RT-PCR test. The positive results were found across four provinces; Benslimane had the highest proportion of positive cases (44.4%), while Settat and Berrechid each had 22.2% of the positives, and El Jadida had one (11.1%) case. No cases were found in Sidi Bennour.

**Conclusion:**

This study presents the first documented case of Bovine Viral Diarrhea Virus infection in Morocco, highlighting its potential for widespread circulation in the country. These results underscore the need for further studies to isolate and characterize circulating strains of BVDV, with the goal of developing effective prevention and control strategies to minimize their negative impacts on cattle health and productivity.

## Introduction

BVDV is a significant viral pathogen of cattle that causes substantial economic losses through reduced productivity, milk yield declines, reproductive failures, morbidity, and mortality [1–3].

BVDV, belonging to the Genus *Pestivirus* in the family *Flaviviridae*, is closely related to classical swine fever virus and border disease virus [4,5]. Among the three recognized species, BVDV-1 and BVDV-2 are most frequently reported and possess considerable genetic diversity, with at least 22 subgenotypes identified for BVDV-1 and 4 subgenotypes for BVDV-2, demonstrating their vast variability [6,7]. A third species, known as HoBi-like Pestivirus-3, has also been identified in some regions [8,9].

BVDV can be divided into two biotypes: Cytopathogenic (CP) and Non-Cytopathogenic (NCP), defined by their effects on cell culture rather than their virulence in animals. NCP biotype predominates naturally, playing an essential role in producing persistently infected (PI) animals, while the CP variant often emerges during persistent infections, with mucosal disease being reported [10–12].

Clinically, the symptoms of BVDV infection vary considerably and range from subclinical cases to severe respiratory, digestive, and reproductive disorders [2]. Intrauterine infections may lead to abortions, stillbirths, congenital defects, or the birth of PI calves, which spread the virus in their herds throughout their lives, furthering viral propagation within herds [13–15].

Diagnostic techniques have dramatically advanced over the past decades. Although virus isolation was once the standard approach, it proved too time-consuming and laborious. Serological methods, such as ELISA and virus neutralization, have improved detection; however, molecular assays, including RT-PCR, have become the gold standard due to their sensitivity and specificity [16,17]. Today, antigen capture ELISA and RT-PCR are widely utilized for the diagnosis of BVDV infections in both clinical and research settings [17,18].

Serological evidence confirms the widespread nature of BVDV infection in Morocco. One investigation conducted between 2018 and 2019 found a prevalence rate of 56.1% among unvaccinated cattle [18], while our most recent nationwide survey (December 2023, February 2024) demonstrated a 25% prevalence across various regions [19]. Despite this evidence, no molecular or antigenic studies have yet been conducted in Moroccan cattle, representing a critical knowledge gap.

This study seeks to fill an existing knowledge gap by conducting the first antigenic and molecular investigation of BVDV in Morocco. Antigen-capture ELISA and real-time RT-PCR assays were employed in this study to detect and confirm BVDV infections in cattle exhibiting respiratory or reproductive disorders in Morocco. This research provided definitive proof of BVDV infection and laid the groundwork for future studies on virus isolation, genetic characterization, and epidemiological surveillance of circulating BVDV strains in Morocco.

## Materials and Methods

### Ethical clearance

All samples were collected as part of routine veterinary supervision activities undertaken by the authorities, and no other sampling or activities were conducted for research purposes. All animal research conducted in this study was carried out in accordance with international standards, as outlined in various scientific references and in the WOAH Manual of Diagnostic Tests and Vaccines for Terrestrial Animals (World Organization for Animal Health, 2024).

### Study area

**Figure 1.**
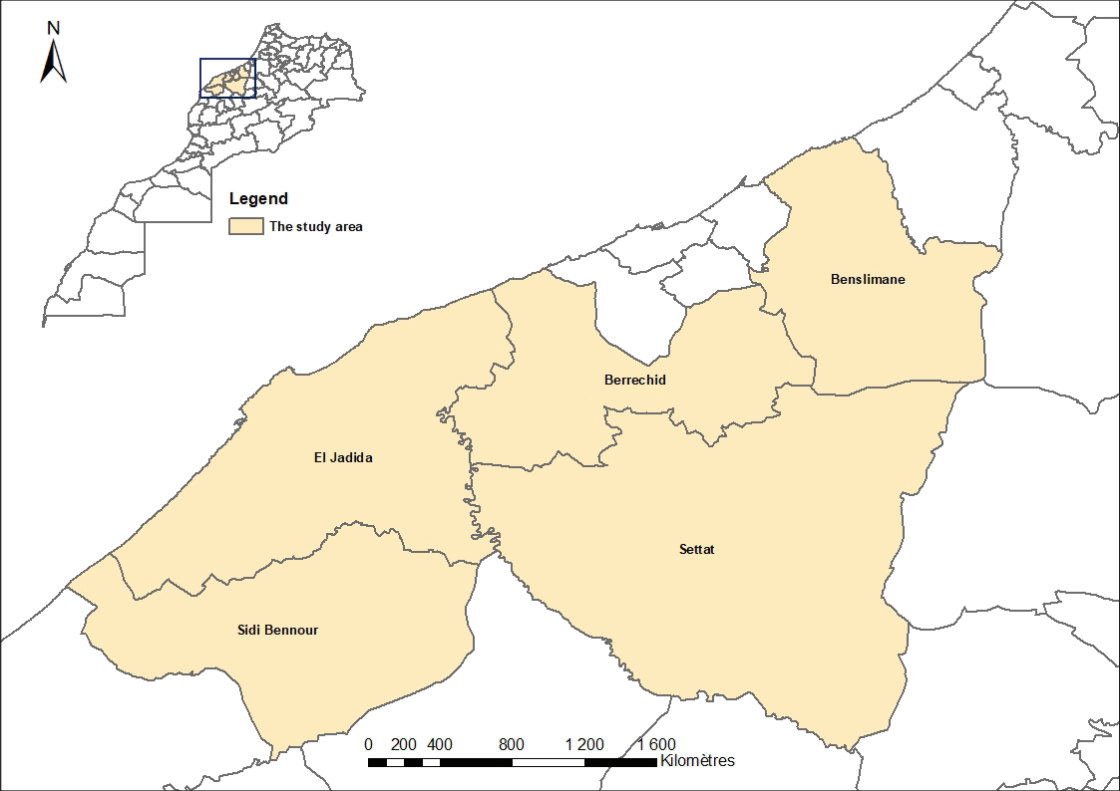
The map illustrates the study area within the Casablanca-Settat region in Morocco; it emphasizes the administrative provinces of El Jadida, Sidi Bennour, Settat, Benslimane, and Berrechid. The inset map shows the location of the region in Morocco.

This study was carried out from January 2021 to June 2025 at the Regional Laboratory of Analysis and Research in Casablanca. A total of 500 serum and whole blood samples from cattle in the Casablanca–Settat region of Morocco (Fig. 1) were gathered for testing at this laboratory. Animals were collected from five provinces (El Jadida, Settat, Sidi Bennour, Berrechid, and Benslimane), all of which are known for having high livestock densities. The Casablanca-Settat region in Morocco serves as a key center of its dairy industry, housing large-scale dairy and beef farms with well-managed cattle that contribute significantly to milk production. This region offers a higher opportunity for identifying BVDV detection, as well as understanding the transmission dynamics due to the higher density of cattle.

### Methodology

The study’s cattle originated from herds that exhibited complications with reproduction and respiratory disease. Hence, the sampling method was purposive rather than random. Animals presenting clinical signs of BVDV infection, such as weakness, fever, diarrhea, stunted growth, or neurological signs like tremors and incoordination, were prioritized for sampling. For this work, all animal sampling was exclusively undertaken by official veterinarians as part of routine diagnostic procedures in full accordance with national regulations and international standards.

Using Vacutainer tubes without an anticoagulant, serum samples were collected. For whole blood samples, tubes containing EDTA were used. All samples were then transported to the Regional Laboratory of Analysis and Research in Casablanca, where they were stored at −20°C until analysis.

Serum and blood samples were collected from each of the five hundred cattle for the study. Serum samples were then subjected to an IDEXX antigen-capture ELISA to test for BVDV antigens, while leukocyte fractions from whole blood samples underwent real-time PCR analysis to detect the genome of BVDV.

### Antigen ELISA

For the detection of BVDV antigens in serum samples according to the manufacturer’s instructions, an ELISA kit known as the IDEXX BVDV Ag/Serum Plus was utilized. Absorbance was measured at 450 nm using a BioTek ELx800 Reader, following all outlined protocols. For the evaluation of validity, positive and negative controls were included, and the percentage inhibition was calculated by subtracting the mean absorbance values of the controls from the sample absorbance values. As per the kit protocol, samples were considered negative when their differences fell below 0.3 and positive when their variance exceeded this threshold.

### RNA extraction

To prevent contamination, all RNA extraction processes were performed within a microbiology cabinet. Use of the NucleoSpin Virus Mini Kit (MACHEREY-NAGEL^®^, Düren, Germany) allowed for the direct extraction of viral RNA from blood samples following the manufacturer’s protocols. A suspension of 150 μl of the sample, prepared by mixing 600 μl of RAV1 lysis buffer and carrier RNA, was incubated at 70°C for 5 min. Next, 600 μl of absolute ethanol was added, and the mixture was transferred to a silica column for centrifugation. According to the provided system, the sample was centrifuged and washed multiple times. The RNA was stored at −80°C after initial Elution in 50 µl of nuclease-free water.

### TaqMan RT-PCR amplification

TaqMan Real-time PCR experiments are conducted in accordance with those described by Hoffmann et al. in 2006 [16]. Primers BVDV190F and V326, along with TaqMan R probe TQ-Pesti, were used to amplify 208 bp from the 5’ UTR region of BVDV. To ensure detection of BVDV-1, BVDV-2, and HoBi-like Pestivirus strains, as indicated in Table 1, primer and probe sequences were selectively degenerated.

**Table 1.** Primers and probe used for BVDV detection and endogenous β-actin internal control in duplex real-time RT-PCR.

Target	Primer/Probe name	Sequence (5′–3′)
BVDV (5′UTR)	BVD 190-F	GRA-GTC-GTC-ART-GGT-TCG-AC
	V326-R	TCA-ACT-CCA-TGT-GCC-ATG-TAC
	TQ-pesti-P	FAM-TGC-YAY-GTG-GAC-GAG-GGC-ATG-C-TAMRA
β-actin (endogenous control)	ACT_F_1005–1029	CAGCACAATGAAGATCAAGATCATC
	ACT_R_1135–1114	CGGACTCATCGTACTCCTGCTT
	ACT_P_1081–1105	VIC-TCGCTGTCCACCTTCCAGCAGATGT-TAMRA

### Endogenous target

ACT_F_1005–1029 (5’-CAG CAC AAT GAA GAT CAA GAT CAT C-3’), ACT_R_1135–1114 (5’-CGG ACT CAT CGT ACT CCT GCT T-3’), and ACT_P_1081–1105 (VIC-TCG CTG TGT CCA CCT TCC AGC AGA TGT-TAM RA) [20] are the sequences of the two primers and the probe used in the duplex real-time RT-PCR assays (Table 1).

### Reaction mixture

For every TaqMan real-time RT-PCR assay, a final volume of 25 µl was prepared. For each TaqMan RT-PCR assay consisting of 0.8 µM forward primer BVD 190-F (5’-GRA GTC GTC ART GGT TCG AC-3’), 0.8 µM reverse primer V326 (5’-TCA ACT CCA TGT GCC ATG TAC-3’), 0.12 µM probe TQ-pesti (5’-FAM-TGC YAY GTG GAC GAG GGC ATG C-TAMRA-3’), and 1× Luna universal probe master mix (New England Biolabs) (Tables 1, 2). A concentration of each primer of 2.5 µM with 1.25 µM of probe was added to the Pre-Mix-Beta-Actin [20]. The reaction volume was adjusted by adding nuclease-free water after the enzyme mixture (Taq DNA polymerase and reverse transcriptase) included in the Luna^®^ kit was introduced at a final concentration of 1×. For all the tests, a total reaction volume of 25 µl was achieved by combining 20 µl of the master mix with 5 µl of the RNA template (Tables 2).

**Table 2.** <span lang="en-GB">Reaction mixture composition for duplex real-time RT-PCR (BVDV + β-actin).</span>

Component	Volume per reaction	Final concentration
Luna^®^ Universal Probe One-Step Reaction Mix (2×)	12.5 µl	—
Luna^®^ WarmStart^®^ RT Enzyme Mix (20×)	1.0 µl	—
Forward primer BVD 190-F (20 µM)	1.0 µl	0.8 µM
Reverse primer V326-R (20 µM)	1.0 µl	0.8 µM
Probe TQ-pesti (3 µM)	1.0 µl	0.12 µM
Forward primer ACT_F_1005–1029 (20 µM)	1.0 µl	0.5 µM
Reverse primer ACT_R_1135–1114 (20 µM)	1.0 µl	0.5 µM
Probe ACT_P_1081–1105(3 µM)	1.0 µl	0.12 µM
RNase-free water	0.5 µl	—
RNA template	5.0 µl	—
Final reaction volume	25.0 µl	—

### Cycling conditions

The thermal cycling was performed on a QuantStudio 5 Real-Time PCR System by Applied Biosystems (Thermo Fisher Scientific). Our protocol begins with 10 min of reverse transcription in which the temperature was maintained at 55°C, then the temperature increased to 95°C for a further 10 min to activate the enzymes. The amplification was performed for 45 cycles of denaturation at 95°C for 15 sec and then at 60°C for 1 min for annealing/extension.

### PCR validation

Positive, negative, and internal controls were routinely included in each PCR run to ensure the reliability of the results. Our internal beta-actin control served to verify the integrity of extracted RNA while ruling out the possible presence of PCR inhibitors in samples. At each run, a positive control was required to produce a clear amplification signal, while the negative controls (without template control) should remain free from any form of amplification. Results were only accepted as valid once these conditions had been fulfilled. Using the set criteria above, samples with cycle threshold (Ct) values below 40 were considered positive, while those above the limit were considered negative.

### Statistical method

The prevalence of BVDV in each province was calculated as the proportion of positive samples to the total number of samples collected and tested in that province. To determine the reliability of these estimates, binomial exact 95% CI were calculated for each of the prevalence values. Additionally, to further elucidate the distribution of infections within the area, the contribution of each province to the total positive cases was determined. This was determined as the proportion of positive samples collected from a particular province to the total number of positive samples collected and reported in the dataset.

## Results

There is a complete absence of literature regarding the existence of BVDV in cattle under clinical observation in Morocco. This is the first instance of its direct detection using antigen-capture ELISA in conjunction with RT-PCR. Out of 500 serum samples tested for BVDV antigens by ELISA, 1.8% (9/500) tested positive, with an OD450 value ranging between 0.56 and 0.91 (mean 0.66). Subsequent RT-PCR analysis confirmed the presence of BVDV RNA with Ct values between 27.4 and 35.4 (mean 30.3) (Tables 4). The concordance between ELISA and RT-PCR results illustrates the reliability of this diagnostic approach, with RT-PCR serving as a validator of serological findings and validating the detection of BVDV infection. In this study, virological detection of BVDV in suspected Moroccan cattle with abortion or respiratory problems was successfully achieved using both antigen detection ELISA and RT-PCR. A total of 1.8% of the tested cattle showed evidence of infection, as identified by these two methodologies.

**Table 4.** Summary of ELISA OD values and RT-PCR Ct values for BVDV-positive samples.

Sample	ELISA OD value (OD450)	RT-PCR Ct value
Sample-01	0.72	27.4
Sample-02	0.85	28.1
Sample-03	0.61	30.7
Sample-04	0.77	31.3
Sample-05	0.87	28.0
Sample-06	0.56	34.5
Sample-07	0.91	27.9
BVDV-08	0.75	29.8
BVDV-09	0.59	35.4

Geographic distribution of BVDV-positive cases within Casablanca–Settat region varied across provinces. Benslimane Province had the highest rate of positive cases (44.44%; 4/9), followed by Settat and Berrechid with two cases each (22.22%; 2/9). El Jadida reported one case (11.11%; 1/9); no positive cases were identified in Sidi Bennour Province (Tables 3).

**Table 3.** BVDV prevalence by Province in the Casablanca-Settat region.

Province	No. of samples tested	No. of positives	Prevalence (95% CI)	% of positives (*n* = 9)
Benslimane	95	4	4.2% (1.6–10.3)	44.4%
Berrechid	103	2	1.9% (0.5–6.8)	22.2%
El Jadida	98	1	1.0% (0.2–5.6)	11.1%
Settat	94	2	2.1% (0.6–7.4)	22.2%
Sidi Bennour	110	0	0.0% (0.0–3.4)	0.0%

## Discussions

Comparing these results with previous research is challenging due to the limited body of knowledge on BVDV infection in Moroccan cattle, particularly since earlier research primarily focused on seroprevalence rather than direct viral detection. A seroepidemiological study conducted between 2018 and 2019 demonstrated a 56.1% seroprevalence of BVDV among unvaccinated cattle, as determined by ELISA antibody testing, providing confirmation of its endemic status [21]. Recently, our research group conducted an extensive serological survey from December 2023 to February 2024, and, using antibody ELISA, identified a seroprevalence of 25%, indicating extensive circulation of BVDV infection in several Moroccan regions [19]. The same research team is now focusing on cattle in the Casablanca-Settat region that are afflicted with respiratory or reproductive problems to gain a more comprehensive understanding of BVDV epidemiology in Morocco.

Emphasizing molecular studies, the only previous study done in Morocco focused on sheep Border Disease Virus (BDV). This study, which integrated serological and molecular methods, did not identify any PI animals using RT-PCR on seronegative samples, confirming that BDV in Morocco is indeed endemic [22].

The incidence rate of 1.8% for BVDV correlates with the antigenic prevalence rate reported in Konya, Turkey [23], but it is considerably lower than rates reported in other studies. For instance, in Iran, a study using antigen capture ELISA reported rates of infection of 17.9% and 20.48% for RT-PCR testing of the same sample [24]. Moreover, studies in several provinces in Northern Turkey indicated a prevalence of 28.57% in aborted fetuses [25].

This disparity might be attributed to differences among the sample populations. In Morocco, the study focused on cattle exhibiting probable signs of BVD, primarily of reproductive or respiratory nature. On the other hand, the Iranian studies focused on aborted fetuses believed to be infected, given their association with reproductive failure and their elevated prevalence.

The presence of BVDV in Casablanca–Settat and Provinces may be attributed to their primary focus of agricultural activities, with intensified cattle farming and more frequent animal movements facilitating virus transmission and increasing the chances of surveillance detection. Moreover, the lower prevalence of BVDV in Morocco suggests that there is more effective herd management, improved biosecurity, or more effective vaccination protocols.

The unexplained absence of DNA from the Bovine Viral Diarrhea Virus from abortion cases leaves the great possibility of other pathogens being present. In Morocco, abortive cases in cattle have been attributed to more than one pathogen, including *Neospora caninum*, *Leptospira* spp., *Coxiella burnetii,* and even bovine herpesvirus type 4 [26,27]. In these cases, the respiratory problems observed in cattle with a BVDV-deficient genome in Morocco may be attributed to bovine respiratory syncytial virus, parainfluenza-3 virus, and bovine rhinotracheitis/infectious pustular vaginitis viruses [21,28]. These findings underscore the importance of conducting more comprehensive differential diagnoses and the subsequent need for targeted and effective diagnostic and control measures to address the pathogens affecting the respiratory and reproductive systems of cattle in Morocco. More extensive investigations should be done, focusing on primary pathogen detection to better define and understand the primary agents of many negative cases.

The recorded instances of BVDV positivity in the Casablanca-Settat region suggest, albeit with varying intensity across different provinces, that the virus is indeed circulating at the regional level. The number of instances noted within the Benslimane province suggests that there may be some special localized conditions that may enhance the transmission of the virus, such as intensive cattle farming, trade, and herd management practices. The detections reported in Settat, Berrechid, and El Jadida suggest that the virus is widespread in this region. However, the absence of positive detections in Sidi Bennour may indicate that circulation levels are lower than expected. Nevertheless, this result could still be due to sampling bias, lower herd exposure, or chance. Overall, these results highlight the significant differences in the province-specific distributions of BVDV and underscore the importance of provincial surveillance in capturing local epidemiological dynamics and accurately diagnosing local epidemic states.

The uneven distribution of BVDV-positive cases across various regions of Casablanca-Settat indicates the potential for BVDV to spread unevenly at the regional level between different provinces. Higher case numbers in Benslimane could be attributed to the higher density of cattle, differences in herd management, or increased trade; in contrast, Settat, Berrechid, and El Jadida showed widespread BVDV infection in their regions. The BVDV results for Sidi Bennour, due to the relatively few positive detections, lower exposure, or sampling, are significantly weaker and should be interpreted with caution. The detection of BVDV in the Casablanca-Settat region highlights the urgent need for province-level control and integrated national animal health policies to ensure the coordinated and long-term sustainable management of BVDV. BVDV continues to significantly undermine the health and productivity of the Moroccan cattle herd, as well as the reproductive performance of cattle, rural incomes, and ultimately, rural livelihoods.

## Conclusion

In this research, detecting BVD antigen and genome in Moroccan cattle herds with respiratory and/or reproductive problems suggests active circulation of the virus in the population. These findings indicate that BVDV poses significant public health risks to the cattle population in Morocco, and the results suggest that BVDV is likely widely distributed throughout the country. There is also a considerable deficiency in knowledge of genetic diversity, pathogenicity, and epidemiological patterns of closely related currently circulating strains. Understanding the strains of BVDV in Morocco will involve thorough research, including isolating and sequencing the virus. With this understanding, effective tissues will incorporate prevention and control measures, such as vaccination programs tailored to local epidemiological conditions. Additionally, improvements in herd management and biosecurity will help limit the spread of the virus. Note, too, that BVDV isn’t the sole agent responsible for respiratory and reproductive problems in cattle; therefore, research on other infectious agents associated with respiratory or abortive syndromes must also be performed to gain an in-depth knowledge of cattle health challenges in Morocco. By considering other pathogens alongside BVDV as disease controllers in tandem, disease losses associated with multifactorial disease complexes will be reduced, thereby improving both the health and productivity of Moroccan herds.

## List ofabbreviations

BVD, Bovine viral diarrhea; BVDV, Bovine viral diarrhea virus; CP, Cytopathogenic; ELISA, Enzyme-linked immunosorbent assay; NCP, non-cytopathogenic; RT-PCR, Reverse transcriptase-Polymerase chain reaction; UTR, untranslated region; μl, microliter; min, minutes; sec, seconds.
